# CHO-produced RBD-Fc subunit vaccines with alternative adjuvants generate immune responses against SARS-CoV-2

**DOI:** 10.1371/journal.pone.0288486

**Published:** 2023-07-14

**Authors:** Sedthawut Laotee, Methawee Duangkaew, Araya Jivapetthai, Kittipan Tharakhet, Papatsara Kaewpang, Eakachai Prompetchara, Supaporn Phumiamorn, Sompong Sapsutthipas, Sakalin Trisiriwanich, Thitiporn Somsaard, Sittiruk Roytrakul, Parichat Duangkhae, Boonsri Ongpipattanakul, Patanachai Limpikirati, Natapol Pornputtapong, Wanatchaporn Arunmanee

**Affiliations:** 1 Department of Biochemistry and Microbiology, Faculty of Pharmaceutical Sciences, Chulalongkorn University, Bangkok, Thailand; 2 Center of Excellence in Vaccine Research and Development (Chula Vaccine Research Center, Chula VRC), Faculty of Medicine, Chulalongkorn University, Bangkok, Thailand; 3 Department of Laboratory Medicine, Faculty of Medicine, Chulalongkorn University, Bangkok, Thailand; 4 Integrated Frontier Biotechnology for Emerging Disease, Chulalongkorn University, Bangkok, Thailand; 5 Institute of Biological Products, Department of Medical Sciences, Ministry of Public Health, Nonthaburi, Thailand; 6 Functional Proteomics Technology Laboratory, National Center for Genetic Engineering and Biotechnology, National Science and Technology for Development Agency, Pathumthani, Thailand; 7 Viral Vaccine Unit, Biologics Research Group, Research and Development Institute, The Government Pharmaceutical Organization, Bangkok, Thailand; 8 Department of Food and Pharmaceutical Chemistry, Faculty of Pharmaceutical Sciences, Chulalongkorn University, Bangkok, Thailand; 9 Center of Excellence in Cancer Cell and Molecular Biology, Faculty of Pharmaceutical Sciences, Chulalongkorn University, Bangkok, Thailand; Emory University, UNITED STATES

## Abstract

Subunit vaccines feature critical advantages over other vaccine platforms such as stability, price, and minimal adverse effects. To maximize immunological protection of subunit vaccines, adjuvants are considered as main components that are formulated within the subunit vaccine. They can modulate adverse effects and enhance immune outcomes. However, the most suitable formulation providing the best immunological outcomes and safety are still under investigation. In this report, we combined recombinant RBD with human IgG_1_ Fc to create an RBD dimer. This fusion protein was expressed in CHO and formulated with alternative adjuvants with different immune activation including Montanide ISA51, Poly (I:C), and MPLA/Quil-A^®^ as potential vaccine candidate formulations. Using the murine model, a potent induction of anti-RBD IgG antibodies in immunized mice sera were observed. IgG subclass analyses (IgG_1_/IgG_2a_) illustrated that all adjuvanted formulations could stimulate both Th1 and Th2-type immune responses in particular Poly (I:C) and MPLA/Quil-A^®^, eliciting greater balance. In addition, Montanide ISA51-formulated RBD-Fc vaccination provided a promising level of neutralizing antibodies against live wild-type SARS-CoV-2 *in vitro* followed by Poly (I:C) and MPLA/Quil-A^®^, respectively. Also, mice sera from adjuvanted formulations could strongly inhibit RBD:ACE2 interaction. This study offers immunogenicity profiles, forecasted safety based on Vaccine-associated enhanced disease (VAED) caused by Th1-skewed immunity, and neutralizing antibody analysis of candidates of RBD-Fc-based subunit vaccine formulations to obtain an alternative subunit vaccine formulation against SARS-CoV-2.

## Introduction

The outbreak of novel coronavirus (SARS-CoV-2) has increasingly become a global threat to humanity. Since the initial outbreak in the city of Wuhan in China’s Hubei province in January 2020, there has been more than 700 million confirmed cases with nearly 7 million deaths globally [1]. This novel pathogen, the cause of COVID-19, causes fever, severe respiratory illness, and pneumonia. A promising approach to control its spread and prevent future outbreaks is the effective vaccination against SARS-CoV-2. To date various types of vaccines such as mRNA, viral vector, protein, and DNA-based vaccines have been shown to induce a strong neutralizing antibody response in animal models as well as humans [2–8]. Two mRNA-based and two adenovirus-based vaccines have been approved by the FDA and EMA whereas more than 50 candidates are still under clinical trials [9]. Many vaccines are still effective against the variants of SARS-CoV-2, however, as more variants continue to emerge, they must still be modified and adapted to tackle future variants. This situation has prompted scientists to continue developing vaccine candidates to prevent future outbreaks and ensure sufficient vaccine availability for low- and middle-income countries.

Immunogen design of SAR-CoV-2 vaccine candidates mostly exploit the spike (S) of SARS-CoV-2. The S proteins of SARS-CoV-2 are abundantly exposed at the surface of the virus and are vital for virus entry into host cells. Hence, the design of novel vaccines should focus on neutralizing the S proteins by antibodies. This homotrimer protein utilizes its two functional subunits; S1 subunit binds to the host cell receptors and S2 subunit is responsible for the fusion of the viral and host cellular membranes. Within the S1 subunit, there is a receptor-binding domain (RBD) that binds to human Angiotensin converting enzyme 2 (ACE2) which mediates viral entry into host cells and stabilizes the prefusion state of the virus. Instead of full-length S proteins, RBD (the S fragment) or its fusion with the Fc domain of human IgG_1_ (RBD-Fc) has been used as a subunit vaccine candidate for SARS-CoV. Several studies demonstrated that RBD and RBD-Fc could elicit potent neutralizing antibodies with no report of complications [10]. Some publications have reported that SARS-CoV-2 spike RBD proteins induced functional antibody response with acceptable safety profile in non-human primates [11, 12]. RBD is an attractive target for developing vaccines against other related coronaviruses (such as MERS-CoV and SAR-CoV) due to promising interference of the binding between RBD and its host cell receptors. This makes RBD-based subunit vaccines a key candidate for SARS-CoV-2 [13, 14]. Recently, many subunit vaccine candidates against COVID-19 have been developed based on SARS-CoV-2 spike RBD e.g., ZF2001, an RBD-dimer (residues 319–537 in tandem repeat) produced in Chinese Hamster Ovary (CHO) cells developed at Anhui Zhifei Longcom Biopharmaceutical along with the Chinese Institute of Microbiology, Academy of Sciences [15]. This candidate was reported to have increased stability and could induce the production of RBD-specific IgG and neutralizing antibody in mouse model. Currently this vaccine candidate is under Phase 3 clinical trial [16]. Another study where dimeric RBD was used as a SARS-CoV-2 vaccine candidate was performed by Sonia Pérez-Rodríguez and their team. They showed that this dimeric RBD fragment linked by intramolecular disulfide bonds has entered Phase 1 clinical trial in 2022. The results indicated promising neutralizing activities without serious side effects [17]. In addition, West China Hospital-Sichuan University has been developing its RBD-based vaccine (residues 319–545) produced in insect cells (baculovirus/SF9) [11]. It was shown that the vaccine candidate is effective in viral protection in non-human primates and is now under Phase 2 clinical trial [18]. Additionally, RBD fused with human IgG Fc fragment (RBD-Fc) has been studied for SARS-CoV-2 vaccine development since it could enhance immunogenicity of RBD and its half-life [19–24]. Currently, at least two RBD-Fc-based vaccine candidates have been investigated in clinical trial studies including AKS-452 and Betuvax-CoV-2 [24, 25] and evidence suggests that RBD-based subunit vaccines are indeed promising candidates.

Adjuvants also play an important role in viral vaccine action by enhancing high titer and long-lived antibody response that can provide long-term protection [26]. Several adjuvants have been used in the development of SARS-CoV-2 subunit vaccines since proteins are not highly immunogenic. Montanide ISA51, used in some MERS vaccines [27, 28], is a water in oil emulsions adjuvant system providing depot effect prolonging subunit vaccine release [29, 30]. Quil-A^®^ and monophosphoryl lipid A (MPLA) have also been used in recent SARS-CoV-2 vaccine development [31]. Quil-A® is a saponin based adjuvant that can induce a strong adjuvant effect to T-dependent as well as T-independent antigens while MPLA is an immunologically active LPS derivative [30, 31]. Polyinosinic-polycytidylic acid (Poly I:C), an dsRNA analog, can activate innate immunity to a viral infection via toll-like receptor 3 (TLR3) which could be beneficial to the action of sub-unit vaccine [32]. Poly I:C has been used in several coronavirus vaccine development [33, 34].

Here we have fused the gene encoding SARS-CoV-2 RBD into a human IgG_1_ Fc in a mammalian expression vector, creating an RBD dimer. The proteins were expressed in CHO cells and secreted into media. Mice were immunized with the purified RBD-Fc with and without adjuvant. Three adjuvants were selected in this study for comparison; Montanide ISA51, Poly (I:C), and MPLA/Quil-A^®^. Immunogenicity and IgG isotypes in sera of mice receiving RBD-Fc were investigated. In addition, measurements of neutralizing antibodies using plaque neutralization test (PRNT) and surrogate virus neutralization test (sVNT) were performed to evaluate the ability that resulting mice antibodies have of neutralizing the virus infection and protecting cells from SARS-CoV-2. As robust immunological outcomes were observed in mice immunized by our RBD-Fc formulated with selected adjuvants, this subunit vaccine candidate is a promising stepping-stone to the development of SARS-CoV-2 vaccines.

## Materials and methods

### Animal and ethics statement

4 to 6-week-old female BALB/c mice used in this study were from Nomura Siam International Co., Ltd. The experimental procedures involving animals were approved by the Committee of Animal Care and Use, Faculty of Medicine, Chulalongkorn University (approval number 006/2563). Animal experiments were conducted in strict accordance with the recommendations of the Ethical Principles and Guidelines for the Use of Animals for Scientific Purposes. Immunization and blood collections were performed under isoflurane anesthesia. All efforts to minimize the suffering of the animals were made throughout the study.

### Cells, viruses, and reagents

ExpiCHO-S cells (Gibco) were grown in ExpiCHO^TM^ expression medium at 37°C, 8% CO_2_, and 125 rpm. African green monkey kidney cells (Vero, ATCC CCL81) were maintained in MEM, 10% fetal bovine serum (FBS), and 1% L-glutamine (37°C and 5% CO_2_) for plaque neutralization assays. The highly pathogenic SARS-CoV-2 was isolated from a clinical specimen from a Chinese patient (hCoV-19/Thailand/74/2020), provided by the National Institute of Health, Department of Medical Sciences, Thailand and was propagated in Vero cells and kept at -70°C.

### Construction of SARS-CoV-2 RBD-Fc in mammalian expression vector for transient expression

The codon-optimized SARS-CoV-2 *rbd* gene (N_334_ –K_529_, accession No. QHD43416.1) with (G_4_S)_3_-linker was synthesized (Twist Bioscience, USA) and inserted into pFUSE-hIgG1-Fc2 (InvivoGen, USA) at *Eco*RI and *Bgl*II restriction sites. The insert was located between IL2 signal sequence and human IgG1 Fc gene. The expression plasmid amplified in *E*. *coli* was extracted for transfection using QIAGEN Plasmid Maxi Kit (Qiagen, Germany). DNA concentration and quality were measured and assessed using Nanodrop One microvolume UV-Vis Spectrophotometer (Thermo Fisher Scientific, USA).

### Transient expression of SARS-CoV-2 RBD-Fc in ExpiCHO-S

Recombinant RBD-Fc was transiently expressed in ExpiCHO-S cells using 40 kDa PEI MAX transfection reagent (Polysciences, USA). In brief, ExpiCHO-S cells were seeded to a final concentration of 0.5 x 10^6^ cell/ml overnight. At the day of transfection, 1 μg/ml of plasmid DNA and 3 μg/ml of PEI MAX were separately diluted in fresh media. Then, diluted PEI MAX was added to the plasmid DNA solution and incubated at room temperature for 10 min. The DNA:PEI MAX complexes were then slowly added to the cell suspension. After five days, the culture was harvested by centrifugation at 5000 g, 4°C for 30 min to remove intact cells and debris. The supernatant was collected and filtered through Steritop Millipore Express PLUS 0.22 μm (Merck, USA).

### Purification of SARS-CoV-2 RBD-Fc

HiTrap™ FF MabSelect™ PrismA column (Cytiva, USA) equipped in ÄKTA Start (GE Healthcare, USA) was equilibrated with the binding buffer (20 mM sodium phosphate buffer, 150 mM NaCl, pH 7.2) at a flow rate of 0.5 ml/min prior to the injection of filtered culture media. After washing the column with the binding buffer, RBD-Fc was eluted by the elution buffer (0.1 M sodium citrate buffer pH 3.0; prepared by mixing 0.1 M citric acid monohydrate with 0.1 M trisodium citrate dihydrate). The protein-containing fractions were collected and neutralized using 1 M Tris-HCl pH 9.0. To remove aggregates, the concentrated sample was further purified via gel filtration chromatography using HiPrep^TM^ 16/60 Sephacryl^TM^ S-200 HR column implemented in ÄKTA Pure (GE Healthcare, USA). The column was previously equilibrated in Phosphate Buffer Saline (PBS; 10 mM Na_2_HPO_4_, 2 mM KH_2_PO_4_, 137 mM NaCl, 2.7 mM KCl, pH 7.4) and continued at a flow rate of 0.5 ml/min after sample injection. The target fractions were pooled and concentrated using Amicon Ultra-4 Centrifugal Filter Unit-10 kDa cutoff (Merck, USA). The recombinant RBD-Fc concentration was measured by Bicinchoninic acid (BCA) assay using Pierce™ BCA Protein Assay Kit (Thermo Fisher Scientific, USA).

### SDS-PAGE and Western blot analysis

Protein samples were resolved by 10% SDS-PAGE followed by Coomassie-blue staining. To observe IgG Fc and SARS-CoV-2 RBD domain in RBD-Fc, the proteins on gels were transferred to Immobilon-NC Transfer Membrane (Merck Millipore, USA). The membranes were then incubated with 5% skim milk (Hardy Diagnostics, USA) in PBS, gently shaking at 4°C for 2 h. After blocking, the membrane was incubated with either 1:5000 diluted anti-Human IgG (Fc specific) peroxidase-conjugated antibody (Sigma-Aldrich, USA) 4°C for 2 h or mouse anti-RBD antibody (R&D Systems, USA) at the ratio of 1:20,000 at room temperature for 1 h. The membrane was then incubated with HRP-conjugated goat anti-mouse antibody (BioLegends, USA) at the ratio of 1:200,000 for 30 min at room temperature. After washing the membranes by PBST three times for 15 min between each step, Immoblion^®^ Forte Western HRP Substrate (Merck, USA) was added to the membrane and incubated at room temperature for 3 min. The immunological complexes were visualized by chemiluminescent ImageQuant (LAS4000) program (GE Healthcare, USA). To determine glycosylation in RBD-Fc, purified RBD-Fc was digested by Endoglycosidase H (Endo H) or PNGase F as per manufacturer’s instructions for “PNGase F Denaturing Protocol” and “Endo H Denaturing Protocol” (New England Biolabs, USA). Deglycosylated proteins were analyzed d by SDS-PAGE followed by Coomassie-blue staining.

### *In vitro* hACE2 binding assay via enzyme-linked immunosorbent assay (ELISA)

Each well of Nunc-Immuno MaxiSorp plates (Thermo Fisher Scientific) was coated with 100 ng/well of human angiotensin-converting enzyme 2 (hACE2; ab151852, Abcam, UK) in 50 mM bicarbonate buffer pH 9.6 at 4°C overnight. The wells were blocked by the blocking buffer (PBS supplemented with 1% bovine serum albumin (BSA)) and incubated at 37°C for 1 h. Subsequently, the wells were washed with PBST three times. Either purified RBD-Fc sample or commercial RBD-Fc (InvivoGen, USA) were 2-fold serially diluted (ranging from 100 to 1.5625 ng/ml as determined by BCA assay) and added to the wells and incubated at 37°C for 1 h. After washing with PBST, 1:1000 diluted anti-Human IgG (Fc specific)-peroxidase antibody was added to the wells, followed by incubation for 1 h at 37°C. TMB chromogen solution (Invitrogen, USA) was added to the plates after washing with PBST. The plates were incubated at room temperature for 20 min and the enzymatic reaction was stopped by adding 1 N H_2_SO_4_. After that, the absorbance at 450 nm was measured by microplate reader CALIOstar (BMG Labtech, Germany).

### Adjuvants and formulations

Prior to immunization, concentration of RBD-Fc was determined using ELISA based on a standard curve of commercial RBD-Fc at different concentrations. Adjuvants including Montanide ISA51 (SEPPIC, France), poly (I:C) (Invivogen, USA), and MPLA/Quil-A® (Invivogen, USA; Invivogen, USA) were used in this study. To formulate RBD-Fc vaccine with adjuvants, 20 μg of purified RBD-Fc was mixed with an equal volume of Montanide ISA51, 50 μg of poly (I:C), or 2 μg of MPLA and 2 μg of Quil-A^®^.

### Mice immunization

Thirty-five female BALB/c mice (4–6 weeks) were divided into 7 groups. Mice were subcutaneously immunized with freshly prepared vaccine formulations at week 0, 3, and 6. PBS formulated with adjuvants were used as a negative control. Sera were collected at pre-immunization day, week 2, 5, and 8 and stored at -20°C. The sera were heat-inactivated at 56°C for 30 min prior to conducting the plaque reduction neutralization test (PRNT) and surrogate virus neutralization test (sVNT).

### IgG titer determination

To evaluate RBD-specific IgG responses from mice sera, 100 ng/well of RBD-histag (GenScript, Singapore) in 50 mM bicarbonate buffer pH 9.6 were coated onto Nunc-Immuno MaxiSorp plates (Thermo Fisher Scientific, USA) at 4°C overnight. Then, the wells were blocked with blocking buffer and incubated at 37°C for 1 h followed by washing with PBST three times. 2-fold serially diluted mice sera in PBS (starting form 1:100) were added to the wells and incubated at 37°C for 1 h. After washing, the wells were incubated with 1:1000 diluted HRP-conjugated secondary antibodies including goat anti-mouse IgG(H+L) (Invitrogen, USA), IgG_1_ (Abcam, UK), or IgG_2a_ (Abcam, UK) at 37°C for 1 h. Unbound antibodies were removed, and the reaction was developed using TMB chromogen solution (Invitrogen, USA) at room temperature for 20 min and then stopped by an equivalent volume of 1 N H_2_SO_4_. The absorbance at 450 nm were measured using a microplate reader CALIOstar (BMG Labtech, Germany). Anti-RBD IgG end-point titers were reported as a reciprocal of a highest dilution factor whose OD_450_ still exceeds a cut-off value. In this study, the cut-off value is defined as an average of OD_450_ from a negative control (blank using PBS instead of mice serum) + 8.042 x standard deviation [35].

### Plaque Reduction Neutralization Test (PRNT)

Prior to PRNT experiment, heat-inactivated serum samples were four-fold serially diluted as 1:100, 1:400, 1:1600 and 1:6400 in MEM supplemented with 2% FBS. The neutralization was performed by mixing equal volumes of the diluted serum and the optimal plaque numbers of SARS-CoV-2 at 37°C in water bath for 60 min. To conduct PRNT experiment, Vero cells were seeded into a 6-well plate at a final concentration of 2 x 10^5^ cells/ml and incubated overnight. After that, the medium was replaced by virus-serum mixture followed by gently mixing every 15 min for 1 h. Then, semisolid medium containing 1.2% methyl cellulose (Sigma Aldrich, USA), 1% of 10,000 units/mL penicillin-10,000 μg/mL streptomycin, and 10% FBS was replaced. Plates were incubated for 6–7 days. Subsequently, the plaques were directly fixed with 10% (v/v) formaldehyde for 30 min. After all culture media were discarded, they were stained by 0.5% crystal violet. The number of plaques formed was counted and the percentage of plaque reduction at 50% were measured. As detection range of sera dilution was limited, PRNT_90_ titer was calculated based on probit equation before conducting analysis.

### Surrogate Virus Neutralization Test (sVNT)

sVNT was performed by using cPass SARS-CoV-2 Surrogate Virus Neutralization Test Kit (Genscript) as per manufacturer’s instructions. Briefly, sera were three-times diluted starting from 1:100 to 1:8100 and mixed with an equal volume of HRP-conjugated RBD then incubated at 37°C for 30 min. PBS was used as a negative control. Each mixture was added into the ACE2-coated plate and incubated at 37°C for 15 min. The plate was washed thoroughly and developed by adding TMB substrate. After 15 min, the reaction was quenched and absorbance in each well was read at 450 nm. Percentage of inhibition of each sample was calculated from (1 – $\frac{\mathrm{signal}\;\mathrm{from}\;\mathrm{sample}}{\mathrm{signal}\;\mathrm{from}\;\mathrm{negative}\;\mathrm{control}}$) x 100.

### Statistical analysis

Due to data non-linearity, IgG titers, PRNT90, and sVNT titer among groups of vaccine treatments for each week were analyzed using Kruskal-Wallis rank test in R packages. Post-hoc analyses were then performed using Dunn Kruskal-Wallis multiple comparison with Benjamini-Hochberg method p-values adjustment.

## Results

### Production of CHO-based SARS-CoV-2 RBD fused with human IgG_1_ Fc domain

**Fig 1:**
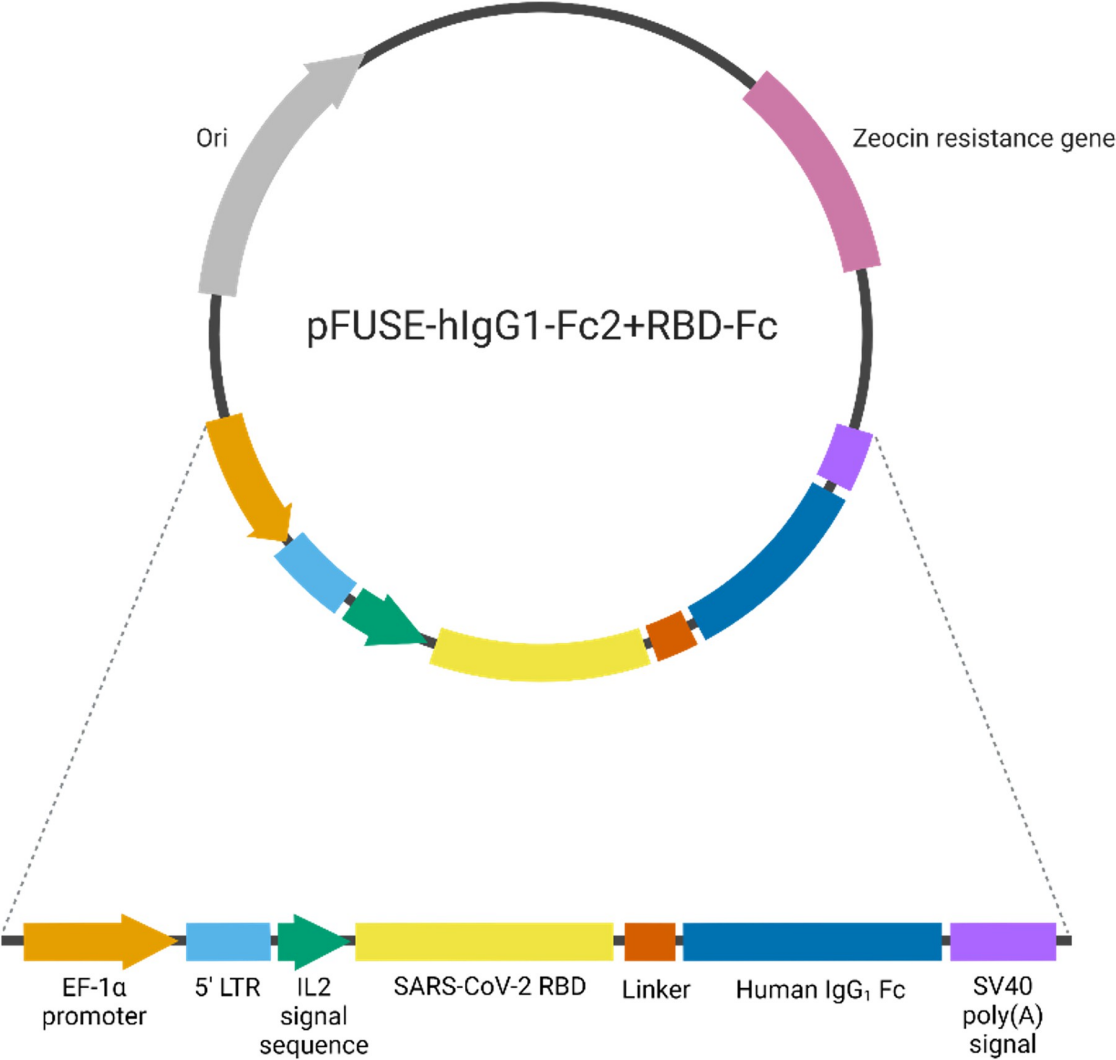
Schematic representation of pFUSE-hIgG1-Fc2 plasmid encoding RBD-Fc.

**Fig 2:**
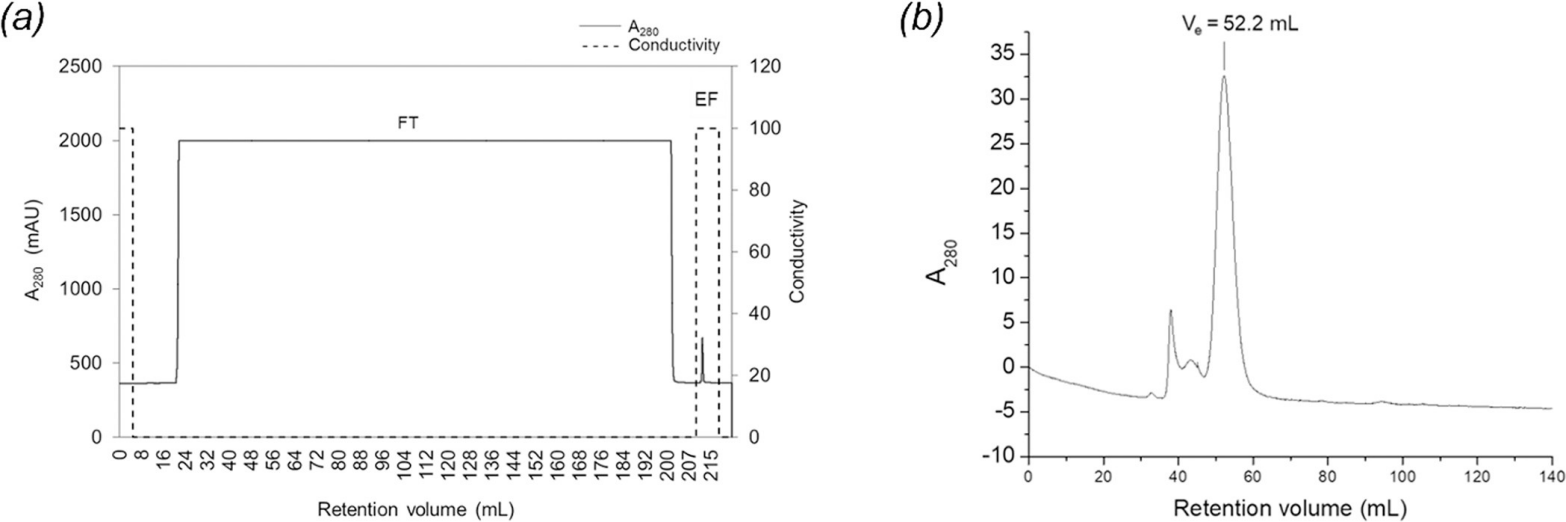
Purification of RBD-Fc by an affinity and size exclusion chromatography. (a) Elution profile of RBD-Fc using a mAbSelect column. (—) A_280_ was displayed as a solid line, while (—) conductivity was plotted as a dash line. (b) Size exclusion chromatogram (SEC) purification graph of RBD-Fc. A_280_ was plotted as a solid black line versus the retention volume.

Recombinant SARS-CoV-2 RBD Fc-fusion protein was produced in ExpiCHO-S cells to achieve similar post-translational modification found in human cells. The coding sequence of RBD was cloned in frame with a human IgG_1_ Fc sequence in pFUSE-hIgG_1_-Fc2 (Fig 1). The recombinant plasmid encoding RBD-Fc was transfected into ExpiCHO-S cells using PEI MAX-mediated method and the cell viability was maintained above 80% during a 5-day expression period. With a IL2 signal sequence located at the N-terminus of RBD-Fc, this signal peptides enabled the secretion of mature proteins resulting in fewer RBD-Fc purification steps. After intact cells and debris removal, the supernatant which contained RBD-Fc was purified by a mAbSelect column which is a protein A column capturing Fc domain. The chromatogram showed that the eluted fractions (EF) containing RBD-Fc were obtained (Fig 2A). Then, the eluted fractions were pooled, neutralized, and further purified by gel-filtration chromatography using HiPrep^TM^ 16/60 Sephacryl^TM^ S-200 HR column to remove aggregation. As displayed in Fig 2B, aggregations were shown as small peaks at approximately 40–45 ml while the RBD-Fc were present at V_e_ = 52.2 ml which contributes to approximately 80% of the relative peak area. Purified RBD-Fc was buffer-exchanged to PBS and its concentration was determined by BCA assay. RBD-Fc yield was 4.2 mg/L of culture.

### ExpiCHO-produced RBD-Fc retained its identity and function

**Fig 3:**
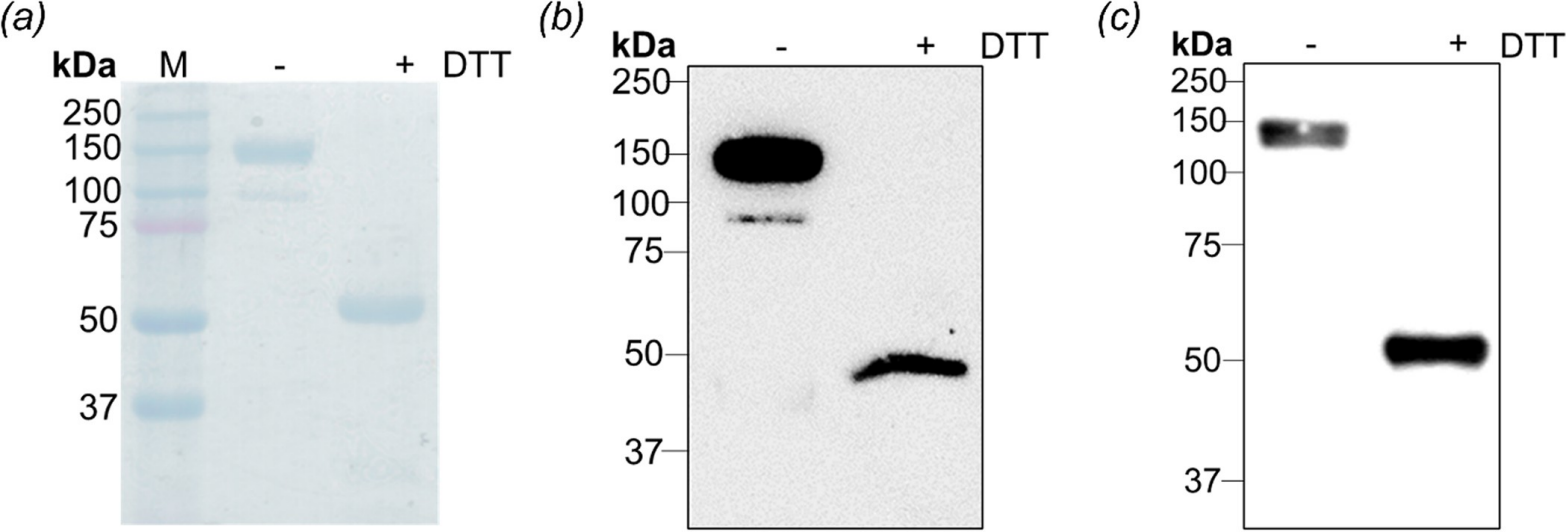
SDS-PAGE and Western blot analyses of purified RBD-Fc. (a) Coomassie blue-stained SDS-PAGE analysis of non-reduced and reduced RBD-Fc. (b, c) Immunoblotting analysis of RBD-Fc using (b) anti-human Fc domain antibody and (c) anti-SARS-CoV-2 RBD antibody as detection antibodies in the presence and absence of DTT.

To evaluate biochemical characteristics of CHO-produced RBD-Fc, purified RBD-Fc was analyzed by SDS-PAGE. The resulting gel showed bands at ~50 and ~150 kDa in reducing and non-reducing conditions, respectively (Fig 3A). According to the theoretical size of RBD-Fc (a 48.8 kDa monomer), the bands at ~50 and ~150 kDa represent monomeric and dimeric forms of RBD-Fc, respectively. The identity of RBD-Fc was confirmed by immunoblotting assay using anti-human IgG1 Fc domain and anti-RBD antibodies. As seen in Fig 3B and 3C, the expected bands were visualized by those antibodies hence recombinant RBD-Fc in this study comprised of RBD and Fc domain. These results suggests that RBD-Fc was successfully expressed and purified in ExpiCHO expression system.

**Fig 4:**
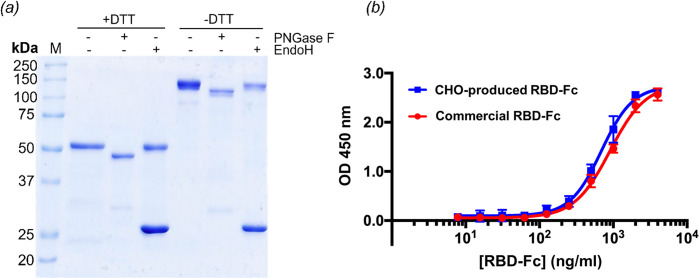
Biochemical characterizations of recombinant RBD-Fc. (a) Deglycosylation analysis of purified RBD-Fc using EndoH and PNGase F evaluated by SDS-PAGE followed by Coomassie-blue staining. (b) Dose-dependent binding of CHO-produced RBD-Fc (blue) and commercial RBD-Fc (red) to soluble human ACE2 as determined by ELISA. Data are presented as a mean of △OD_450_ ± S.D.

Furthermore, to observe the N-linked glycosylation on RBD-Fc, RBD-Fc was digested by Endo H or PNGase F prior to observation of deglycosylated products by Coomassie-blue stained SDS-PAGE (Fig 4A). PNGase F enzyme catalyzes the cleavage of N-linked glycosylation in high mannose, hybrid, and complex glycoforms whereas Endo H enzyme cleaves glycosidic bond of high mannose and hybrid glycoforms. Theoretically, RBD and human Fc were calculated to be 23.3 and 25.6 kDa, respectively. Therefore, the molecular weights of RBD-Fc monomer and dimer was expected to be 48.9 kDa and 97.8 kDa, respectively. Aglycosylated RBD-Fc monomer resulted from PNGase F digestion migrated at the calculated molecular weight of 48.9 kDa. RBD-Fc monomer from untreated and Endo-H treated samples were slightly above 50 kDa as the glycans were not cleaved. In the case of dimeric RBD-Fc, their migration was not running at the expected molecular weight due to the glycosylation and non-reducing conditions. The PNGase F treated RBD-Fc dimers ran faster than untreated and Endo H treated samples. This implies that the glycans of RBD-Fc dimers were only digested by PNGase F, and not by Endo H. This SDS-PAGE analysis indicated that CHO-based RBD-Fc was decorated by complex N-glycosylation. Additionally, the band observed in Endo H treated samples at ~25 kDa is most likely the Endo H enzyme, 29 kDa. A prominent band was observed for Endo H but not PNGase F because the concentration used for Endo H was much higher according to manufacturer’s instructions when these two enzymes were added.

We then determined receptor binding ability of SARS-CoV-2 RBD in RBD-Fc by using human ACE2-dependent ELISA as ACE2 is a receptor for SARS-CoV-2 host-cell internalization and was reported to have a strong binding affinity against SARS-CoV-2 RBD. Using a 96-well plate format, human ACE2-coated wells were incubated with 2-fold serially diluted RBD-Fc starting from 100 ng/ml. Anti-human IgG (Fc specific)-peroxidase antibody was used to detect RBD-Fc followed by addition of TMB chromogen solution to allow enzymatic reaction of peroxidase, and H_2_SO_4_ was used in the final step to stop enzyme reaction. As seen in Fig 4B, the results showed that our RBD-Fc which was purified by size-exclusion column chromatography could bind to human ACE2 in a dose dependent manner similar to commercial RBD-Fc.

The primary structure of RBD-Fc was characterized by peptide mapping using bottom-up LC-MS/MS. RBD-Fc amino acid sequence was used for the database search to sequence and identify the peptide fragments, and 84% sequence coverage was obtained from the LC-MS/MS study, confirming the identity of purified protein as RBD-Fc.

### Mice sera receiving RBD-Fc vaccine elicited potent anti-RBD antibody

**Fig 5:**
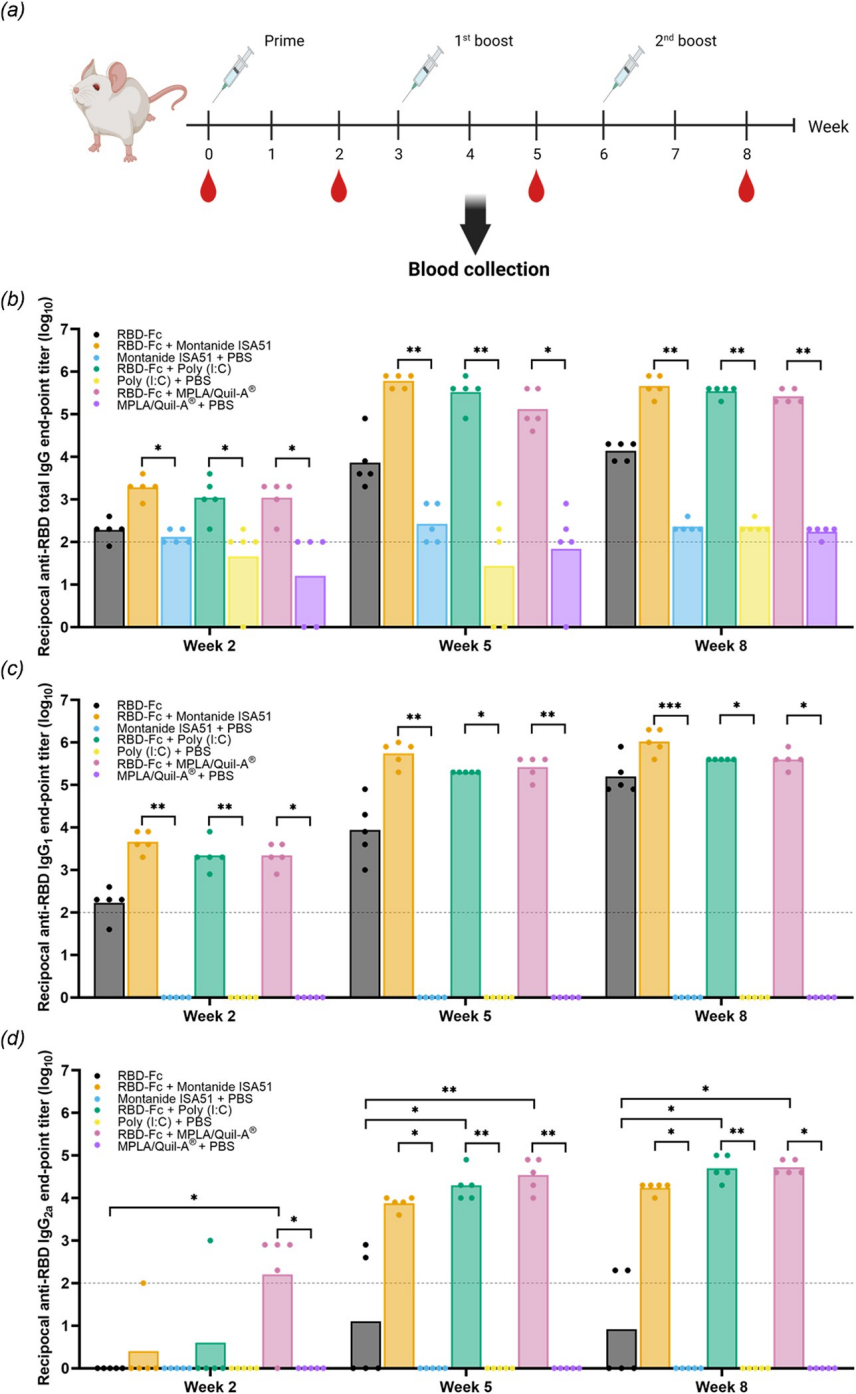
Immunogenicity study and IgG subclasses profile of RBD-Fc formulated with different adjuvants in mice model. (a) Schematic flow chart of the immunization experiment. Reciprocal anti-RBD (b) total IgG, (c) IgG_1_, and (d) IgG_2a_ titers from mice sera collected in week 2, 5, and 8. Each dot represents a different subject. Limit of detection (titer less than 100) was presented in a dash line. The titers were presented as a geometric mean. (*, p<0.05; **, p<0.01; ***, p<0.001).

The immunogenicity of RBD-Fc vaccine in murine model was evaluated using BALB/C mice. A three-dose schedule was applied where each group of mice received different formulations with 3-week intervals between each dose. The seven formulations included one 20 μg RBD-Fc, three 20 μg RBD-Fc with selected adjuvants, and three PBS formulated with adjuvants as negative control groups. In case of formulations with adjuvants, an equal volume of Montanide ISA51, 50 μg of Poly (I:C), and 2 μg of MPLA/Quil-A^®^ were mixed with either proteins or PBS. The samples of mice serum were collected at pre-immunization and two weeks after each immunization; week 0, 2, 5, and 8 (Fig 5A). The level of antibodies against SARS-CoV-2 RBD that were generated in immunized mice was determined by indirect ELISA using commercial RBD-histag as a capture antigen. HRP-conjugated anti-mouse total IgG served as a detection antibody. The results showed that RBD-Fc with adjuvants induced a greater level of anti-RBD total IgG when compared to those in control groups after first immunization, while signals from mice sera receiving RBD-Fc alone were comparable to control groups (Fig 5B). However, in RBD-Fc groups without adjuvants the titer of anti-RBD total IgG increased after each booster administration, yielding a geometric mean titer (GMT) of 13,863 at week 8. In addition, mice sera injected with adjuvanted formulations exhibited promising anti-RBD total IgG titer. The highest GMT after complete vaccination course was obtained from Montanide ISA51 formulation (459,479) followed by Poly(I:C) (348,220) and MPLA/Quil-A^®^ (263,902), respectively. However, the potent total IgG response was not significantly different among adjuvanted sample groups. This result showed that CHO-produced RBD-Fc was immunogenic in mice and that vaccine formulations consisting of adjuvants enhanced its immunogenicity.

RBD-specific IgG subclasses profiles containing IgG_1_ and IgG_2a_ were also examined using similar ELISA procedure that used HRP-conjugated anti-mouse IgG_1_ and IgG_2a_ as detection antibodies. Like specific total IgG titer, RBD-Fc-vaccinated mice sera induced a low level IgG_1_ response, while adjuvated groups exerted higher titers after prime immunization (Fig 5C). At week 8, RBD-Fc with Montanide ISA51 group showed the greatest IgG_1_ response (GMT of 1,080,037), while poly(I:C) and MPLA/Quil-A^®^ formulations exerted lower IgG_1_ titer (400,000 and 406,405, respectively). In contrast to adjuvanted vaccinated groups, mice sera injected by RBD-Fc alone induced the lowest IgG_1_ titer (171,903). As shown in Fig 5D, RBD-specific IgG_2a_ level was significantly different among mice receiving different vaccine formulations. At first immunization, MPLA/Quil-A^®^ formulation induced the highest anti-RBD IgG_2a_ titer, while signals from other groups were mostly undetectable. Mice that received RBD-Fc with MPLA/Quil-A^®^ displayed increasing IgG_2a_ response until week 8 (52,780) which was comparable to result from RBD-Fc with Poly(I:C) group (50,238). On the contrary, Montanide ISA51 formulation showed lower IgG_2a_ level (17,411) and RBD-Fc only vaccinated mice failed to provoke an IgG_2a_ response as IgG_2a_ from 3 of 5 mice sera were below detection range. Collectively, the IgG_1_/IgG_2a_ profile indicated a distinct RBD-specific IgG isotype induction from RBD-Fc vaccine formulations. RBD-Fc only formulation did not induce an IgG_2a_ response. While Montanide ISA formulation exhibited a IgG_1_-bias response, Poly (I:C) and MPLA/Quil-A^®^ formulations showed a greater balance of IgG_1_/IgG_2a_ levels. Fig 5B–5D shows that the RBD-specific antibody levels including total IgG, IgG_1_, and IgG_2a_ were enhanced sharply after receiving the first booster (week 3).

### RBD-Fc-vaccinated mice sera induced strong neutralization activity against live SARS-CoV-2 virus

**Fig 6:**
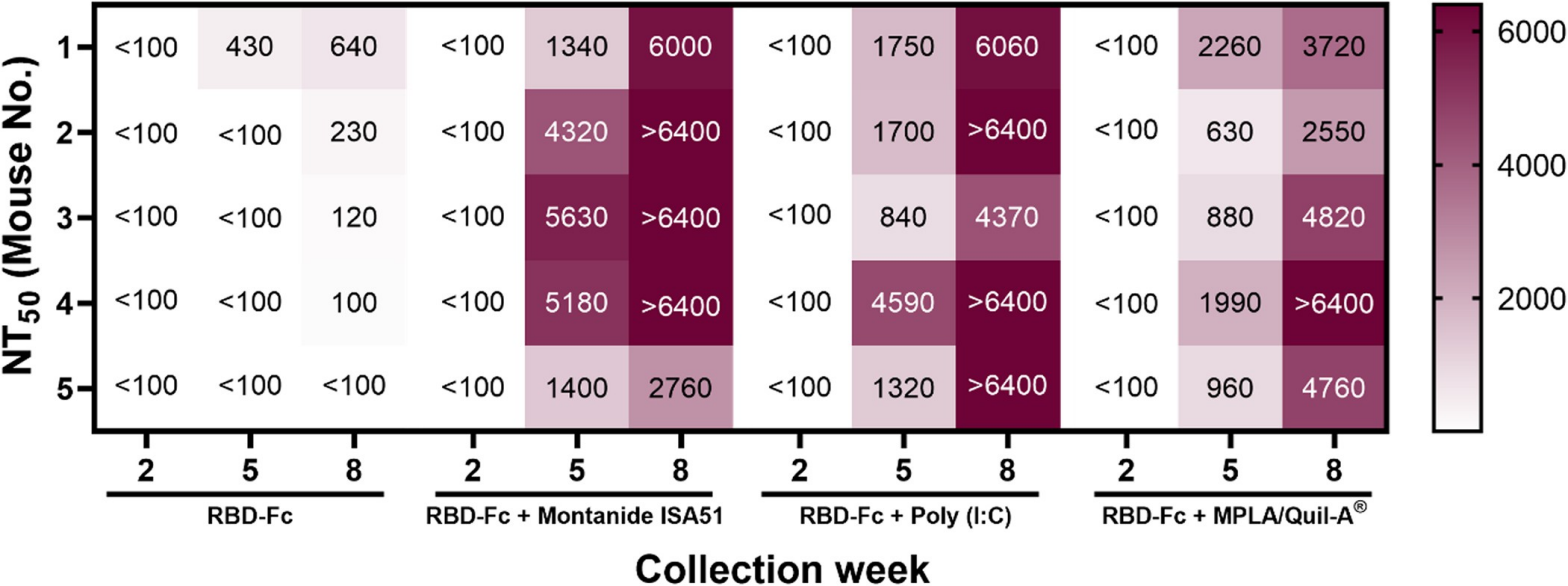
Heat-map of NT_50_ titer of vaccinated mice sera against live SARS-CoV-2 virus. Five BALB/C mice sera receiving different formulations of RBD-Fc vaccine were collected at two-week intervals after each immunization (week 2, 5, and 8) and tested by plaque reduction neutralization assay. Detection range is 100 to 6400.

**Fig 7:**
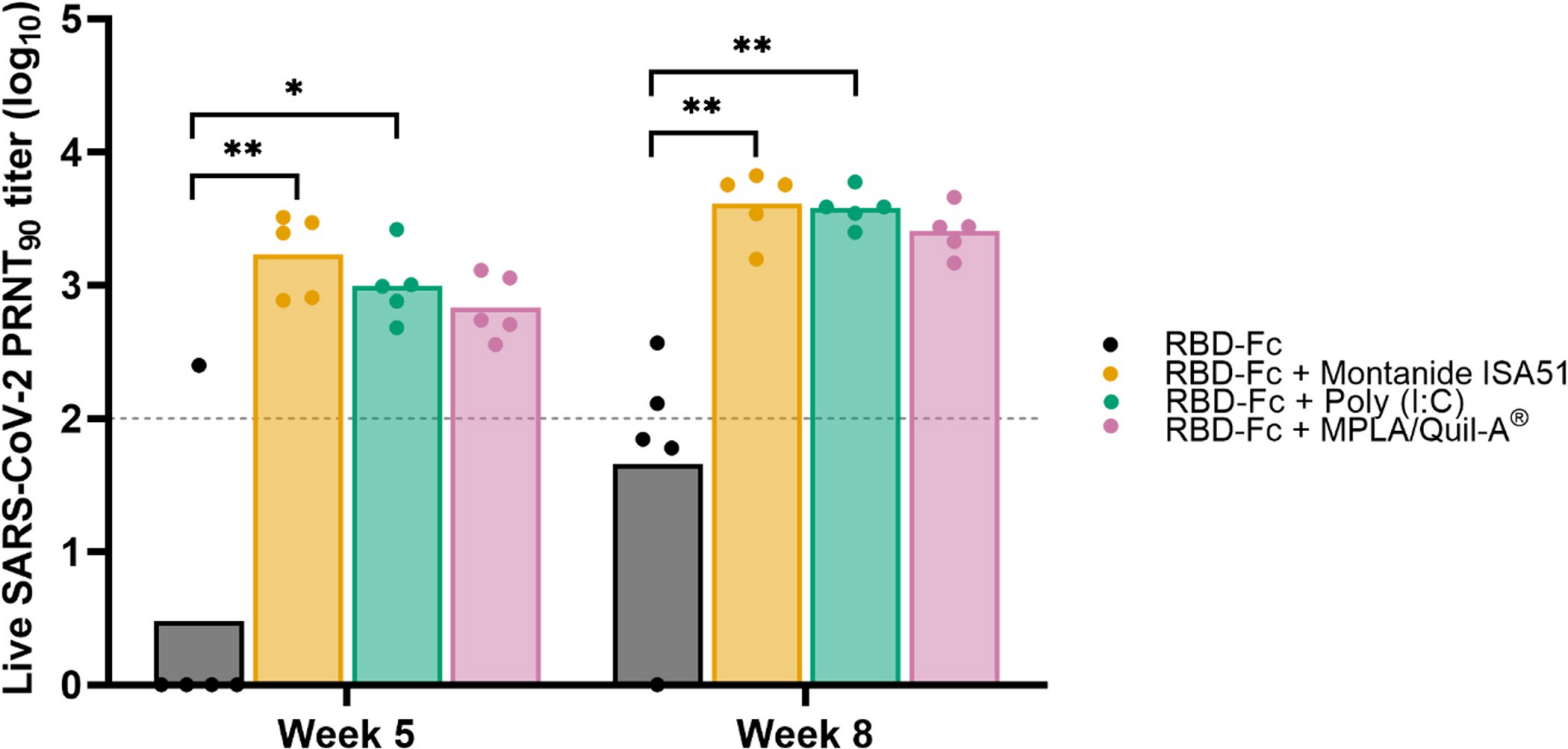
Calculated NT_90_ titer of mice sera against wild-type SARS-CoV-2. Dilution of mice sera collected at week 5 and 8. Each dot represents an individual subject. The titers were displayed as a geometric mean. (*, p<0.05; **, p<0.01; ***, p<0.001).

Neutralizing antibody of mice sera immunized with different RBD-Fc vaccine formulations was evaluated by plaque reduction neutralization test (PRNT). A live wild-type of SARS-CoV-2 was neutralized by heat-inactivated mice sera collected at week 2, 5, and 8 prior to infection of Vero cells. After that, plaques were counted and calculated to obtain NT_50_ which is the highest sera dilution that reduced viral plaques by 50% compared to negative control. As displayed in Fig 6, neutralizing antibody from mice sera from all formulations were undetectable at prime vaccination, but the NT_50_ levels were elevated after first and second booster immunizations. At week 5, mice sera receiving RBD-Fc only immunization had low neutralizing antibodies against the live virus, while titer from other formulations including RBD-Fc with adjuvants were higher. GMT of NT_50_ of RBD-Fc with Montanide ISA51, Poly(I:C), and MPLA/Quil-A^®^ formulation at week 5 were 2,983, 1,722, and 1,191, respectively. Interestingly, 3 of 5 mice immunized with RBD-Fc with Montanide ISA51 or Poly(I:C) elicited superior NT_50_ levels beyond detection range, while MPLA/Quil-A^®^ formulation had only one mouse whose NT_50_ exceeded detection range after receiving a second booster. Conversely, low-level NT_50_ was observed from formulation of RBD-Fc only. Due to detection range limitations, NT_90_ (the determination of maximum serum dilution needed to reduce virus plaque by 90% among Vero cells) was calculated based on probit analysis (Fig 7). The weak signals of the control groups as shown in Fig 5C and 5D indicated that these formulations were not able to induce any detectable amount of anti-RBD IgG in mice. Therefore, these control groups were not included in the experiments shown in Fig 7. Statistical evaluation showed that NT_90_ from mice receiving RBD-Fc with Montanide ISA51 (GMT of 4,111) or Poly(I:C) (3,794) was found to be significantly superior to MPLA/Quil-A^®^ formulation (2,556) and non-adjuvanted RBD-Fc. The results illustrated a noteworthy impact of adjuvants because RBD-Fc alone did not express sufficient neutralizing antibody titers while adjuvanted formulations showed promising neutralizing antibody titers. Furthermore, as NT_90_ from week 5 and 8 were compared, the result revealed a significant difference from adjuvanted groups between first and second booster (*p*-value is less than 0.05 for Montanide ISA51 and MPLA/Quil-A^®^ formulation and 0.001 for Poly(I:C) formulation).

### Adjuvated RBD-Fc vaccine formulation provided a robust inhibition against ACE2:RBD interaction

**Fig 8:**
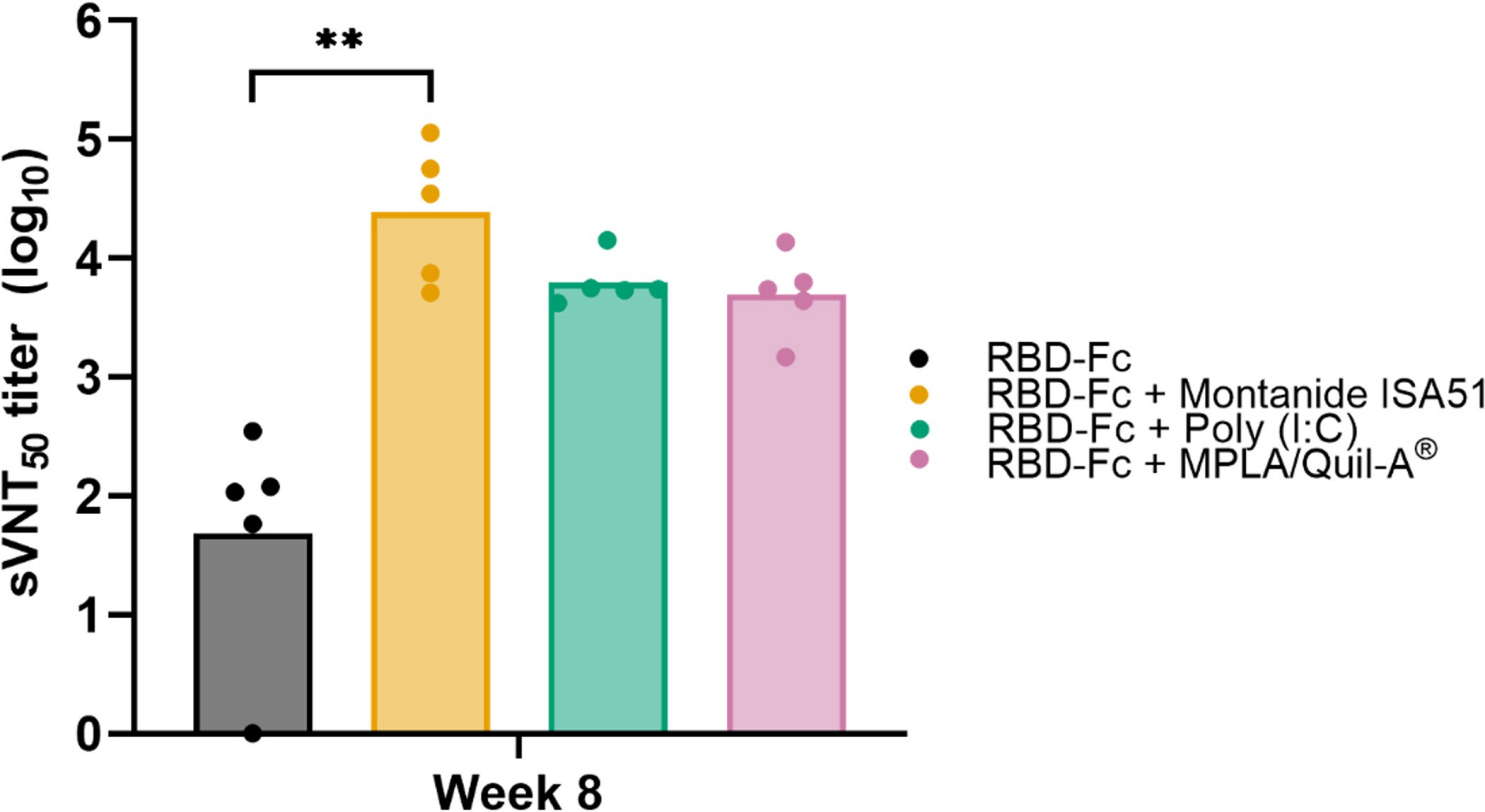
Inhibitory activity against interaction between SARS-CoV-2 RBD and host receptor, ACE2, elicited by immunized mice collected at week 8. Mice sera were analyzed by competitive ACE2-coated ELISA against RBD conjugated with HRP. Titers were shown as a mice sera dilution that inhibited RBD-ACE2 interaction over 50% compared to negative control. Each dot represented individual subjects. The titers were plotted as a geometric mean. (*, p<0.05; **, p<0.01; ***, p<0.001).

Since neutralizing antibodies raised by vaccine candidates aim to inhibit viral host cell entry, we determined inhibitory activity against interaction between wild-type SARS-CoV-2 RBD and human ACE2 from RBD-Fc-vaccinated mice sera by using surrogate virus neutralization test [36]. As a competitive ELISA was performed, the highest mice sera that reduced the signal from RBD:ACE2 interaction by 50% was calculated (sVNT_50_). After a second booster, mice sera receiving RBD-Fc with Montanide ISA51 showed an apparent sVNT_50_ (GMT of 24,416) that was significantly greater than RBD-Fc only group (Fig 8). Furthermore, Poly(I:C) and MPLA/Quil-A^®^ formulations displayed basal level of sVNT_50_ (6,286 and 4,981, respectively). The results illustrated that RBD-Fc with adjuvant could inhibit the interaction of viral RBD and human ACE2. Based on neutralizing antibody level and inhibitory effect, Montanide ISA51 formulation exhibited the best outcome followed by Poly (I:C), and MPLA/Quil-A^®^, respectively.

## Discussion

To date, several types of vaccines such as mRNA, viral vector, and inactivated vaccines against SARS-CoV-2 have been approved for human use such as mRNA-1273, BNT162b2, Ad5-nCoV, AZD1222, and Coronavac. In contrast, only a limited number of subunit vaccines have been approved such as NVX-CoV2373, ZF2001 [9]. However, in order for subunit vaccines to show adequate immune response, adjuvants need to be added. A major advantage of subunit vaccines is its stability and uncomplicated storage conditions resulting in a promising vaccine platform for low-income countries where logistical issues can hinder efficient local vaccine distribution. Therefore, the combination of subunit vaccine with different types of adjuvants that gives the best immunological protection needs to be investigated.

Herein, we established the use of CHO-produced recombinant RBD fused with human IgG_1_ Fc fragment with native structure and function as a promising vaccine candidate; this fusion protein also elicits an elevated immune response. Due to the low immunogenicity of SARS-CoV-2 RBD, several approaches were conducted to enhance the immunogenicity of RBD-based vaccine such as fusion of RBD with nanoparticles, immune booster peptides, and production of RBD multimers [37–40]. Lianpan Dai *et al*. illustrated the use of dimeric RBD vaccine linked via disulfide bonds. The results showed that the RBD-dimer elicited higher IgG titer and neutralizing antibody levels in mice compared to the RBD monomer, suggesting the potential use of dimeric form of RBD as vaccines [15]. We chose Fc-fusion platform to present dimeric RBD through disulfide linkage of Fc fragments. In addition, the Fc fragment has been extensively used to aid the purification process and stability as well as immunopotentiator [41–43].

ExpiCHO-S, a CHO-derived cell, was chosen as the expression host of RBD-Fc due to its many advantages such as ability to aid production, scalability, enhance protein yield, and having desired post-translational modification. This expression system allows the secretion of RBF-Fc to serum-free media, aids purification by affinity chromatography, and ensures achievement of mature proteins. After two-step purification was conducted, the yield of RBD-Fc was around 4.2 mg per liter of culture. Even though low protein yield was obtained in this experiment, a higher protein yield could be achieved by using more effective transfection reagent in transient expression, optimizing culture conditions, and performing stable expression. ExpiCHO-S expression system allows human-like N-glycosylation processes, suggesting the presence of complex glycosylated RBD. Compared to other findings, production of glycosylated RBD is preferable to mammalian expression system since other systems like bacteria, yeast, and insect systems expressed altered glycosylation or no post-translational processes which may contribute to immunogenicity of the vaccine [7, 11, 38, 44]. In addition, the glycosylation pattern between recombinant S proteins produced in mammalian cells and infectious virus S proteins were compared. It demonstrated that the glycans on recombinant S proteins in mammalian cells mimic S proteins derived from infectious virus that were cultured in Vero cells [45]. This ensures that the production of either RBD or S protein in mammalian cells are structurally similar to native virus S proteins. Furthermore, binding analysis of RBD-Fc against human ACE2 by ELISA showed functional activity of our RBD-Fc. Combining these results with the result of primary structure analysis using MS/MS, it was confirmed that CHO-produced RBD-Fc could display native-like properties. This results also were consistent with previous studies where RBD-Fc vaccine obtained from mammalian cell expression system exhibited desired biochemical characteristics [19–21, 24].

Immunogenicity study of RBD-Fc vaccine formulated with different adjuvants showed that RBD-Fc itself induced RBD-specific total IgG antibody, but adjuvanted formulations induced stronger responses. Unlike other RBD-based vaccine candidates containing only RBD fragments, low titers of RBD-specific antibodies were found in the mouse model [7, 11]. This confirmed that Fc-fusion strategy could boost immunogenicity of SARS-CoV-2 RBD. We then assessed anti-RBD IgG subclasses including IgG_1_ and IgG_2a_ to gather more information. One major concern with SARS-CoV-2 vaccine development is vaccine-associated enhanced diseases (VAED). This phenomenon is associated with aberrant type 2 helper T cell (Th2)-type immunity resulting in lung injury by massive eosinophil filtration [46–48]. The occurrence was found in patients immunized with whole inactivated vaccine for measles and respiratory syncytial virus (RSV) and SARS-CoV vaccine studies in mice [49, 50]. To avoid this negative outcome, vaccine candidates that stimulates both Th1 and Th2 with no bias towards Th2 is preferable. In mice, production of IgG_1_ and IgG_2a_ antibody indicates Th2 and Th1 response, respectively [51, 52]. Non-adjuvanted RBD-Fc vaccine formulation induced very low IgG_2a_ levels suggesting Th2-bias responses. Similarly, another finding illustrated that Th2-skewed immunity was provoked by non-adjuvanted RBD-based vaccine candidates, confirming that RBD itself could trigger immune responses towards Th2 immunity [38]. Adjuvants are added to vaccine formulations to not only boost antigen immunogenicity but also to regulate immune responses [53–55]. We found that adjuvanted formulations in this study including Montanide ISA51, Poly (I:C), and MPLA/Quil-A^®^ elicited a distinctive pattern of Th1/Th2 immunity. Even though all adjuvanted formulations provided both RBD-specific IgG_1_ and IgG_2a_ antibodies suggesting the elicitation of Th1 and Th2 responses, RBD-Fc with Poly (I:C) or MPLA/Quil-A^®^ induced greater balance of Th1/Th2 than Montanide ISA51. This could be explained by the similar action mechanism of Poly (I:C) and MPLA that act as a toll-like receptor agonist as opposed to a water-in-oil emulsion based formulation of Montanide ISA51 [56–59]. In addition, we illustrated that adjuvanted formulations strongly elicited potent neutralizing antibodies against wild-type SARS-CoV-2 which further highlighted the importance of adjuvants including Montanide ISA51, Poly (I:C), and MPLA/Quil-A^®^. Compared to Spike-based subunit vaccine candidate, NT_50_ from our RBD-Fc formulated with Montanide ISA51 and Poly (I:C) were comparable to whole spike vaccine candidate (S-2P) [60]. This implied that RBD-based vaccine could elicit sufficient neutralizing antibodies when compared to whole spike candidate. These results were also consistent with other reports of RBD-Fc-based vaccine development that RBD-Fc shows high potency as vaccine candidate against SARS-CoV-2 [17, 19–24].

Currently, there are various adjuvants used in SARS-CoV-2 subunit vaccine development that are in clinical trials such as alum, CpG1018, Matrix-M, Montanide ISA 720, and botulin-based spherical nanoparticles [7, 8, 24, 25, 61, 62]. Although alum has been used in various vaccine formulations, several studies demonstrated that alum could predominantly exhibit bias towards Th2 immune responses [63]. Recent studies on COVID-19 subunit vaccine also demonstrated Th2-bias immune responses based on IgG subclasses and cytokines profile analyses [38, 39, 64]. Another strategy to balance immune responses of Alum is formulation with CpG1018, a toll-like receptor 9 agonist, which has been used in a SARS-CoV subunit vaccine candidate. Alum combined with CpG1018 formulated RBD-based candidate could provide more IgG_2a_ production suggesting a greater Th1 immunity [65]. Also, a SARS-CoV-2 Spike subunit vaccine exhibited a similar phenomenon [60]. Additionally, common side effects including fever, cough, and headache have been reported in Alum-formulated COVID-19 vaccines [7, 62]. Therefore, it would be beneficial to formulate a vaccine using other adjuvants with minimized adverse reactions. Apart from CpG1018, this study showed that Poly(I:C), another TLRs agonist, formulated RBD-Fc vaccines which could induce well-balanced Th1/Th2 response suggesting that using Poly(I:C) provided favorable and sufficient immunological effects. Based on virus-specific antibodies production, Montanide ISA51 exhibited Th2-type immunity favors as well as alum. Another emulsion-based adjuvant, MF59, also triggers immune responses towards Th2-type immunity [66]. However, previous studies illustrated that BALB/c mice showed Th2-bias immune responses [67]. Hence, Th2-polarized immune responses from RBD-Fc with Montanide ISA51 could be reduced, implying balanced Th1/Th2 responses. Moreover, RBD-Fc with Montanide ISA51 vaccine showed potent immune responses in obese mice, suggesting that this formulation is safe and effective for conditioned individual [20]. Another formulation, Montanide ISA720, formulated with CHO-produced RBD-Fc vaccine has entered clinical study, suggesting a promising use of this water in oil emulsion system in combating SARS-CoV-2 [25]. Novavax’s Matrix M is saponin-based particulate adjuvant used in NVX-CoV2373 which passed phase III clinical trial and showed minimal side effects [61]. Similar to our adjuvant candidate, MPLA/Quil-A^®^ has been used in SARS-CoV-2 subunit vaccine and showed great immune responses [31]. On the other hand, convincing levels of both RBD-specific binding antibodies and neutralizing antibodies could not be achieved by using MPLA/Quil-A^®^ compared to other adjuvants, Montanide ISA and Poly (I:C). Additionally, a similar combination of adjuvants such as MPLA/Quil-A^®^ also showed great potential. Juan Shi *et al*. presented MPLA combined with, a plant extract, QS21, formulated with HEK293F-expressed RBD-Fc subunit vaccine showed high antibody titer and neutralizing activities against SARS-CoV-2 [19]. This report supports the use of MPLA/Quil-A^®^ as a candidate for effective adjuvant system. Taken together, we suggest that a CHO-produced RBD-Fc vaccine combined with these selected adjuvants could be a promising alternative vaccination strategy.

## Conclusion

A SARS-CoV-2 subunit vaccine candidate comprising of the fusion of human IgG_1_ Fc with the viral receptor binding domain (RBD) which showed promising immunological correlations in mice model has been proposed. Using CHO-based expression system, purified RBD-Fc was obtained by a two-step purification method and exhibited native-like functions. As adjuvants can modulate vaccine affects differently, three systems including Montanide ISA51, Poly (I:C), and MPLA/Quil-A^®^ were compared. High titer of anti-RBD IgG were observed in adjuvated mice sera. RBD-specific IgG_1_/IgG_2a_ analysis revealed a greater balance of Th1/Th2 immunity induced by Poly (I:C) and MPLA/Quil-A^®^ groups. On the contrary, mice that received Montanide ISA51 plus RBD-Fc elicited the highest neutralizing antibody level against live SARS-CoV-2 determined by PRNT and sVNT assay. These observations offer beneficial information of an alternative SARS-CoV-2 subunit vaccine and choice of adjuvants.

## Supporting information

S1 Raw images Original images underlying all blot or gel results. (PDF)

S1 File Supplementary tables showing minimal data set. (DOCX)
