# Supplementary material for: CHO-produced RBD-Fc subunit vaccines with alternative adjuvants generate immune responses against SARS-CoV-2
Source: PLoS One. 2023 Jul 14;18(7):e0288486. doi: 10.1371/journal.pone.0288486 (PMC10348575; doi:10.1371/journal.pone.0288486)
Supplement: S1 Raw images — (PDF) [file pone.0288486.s001.pdf]

**Figure 3a**

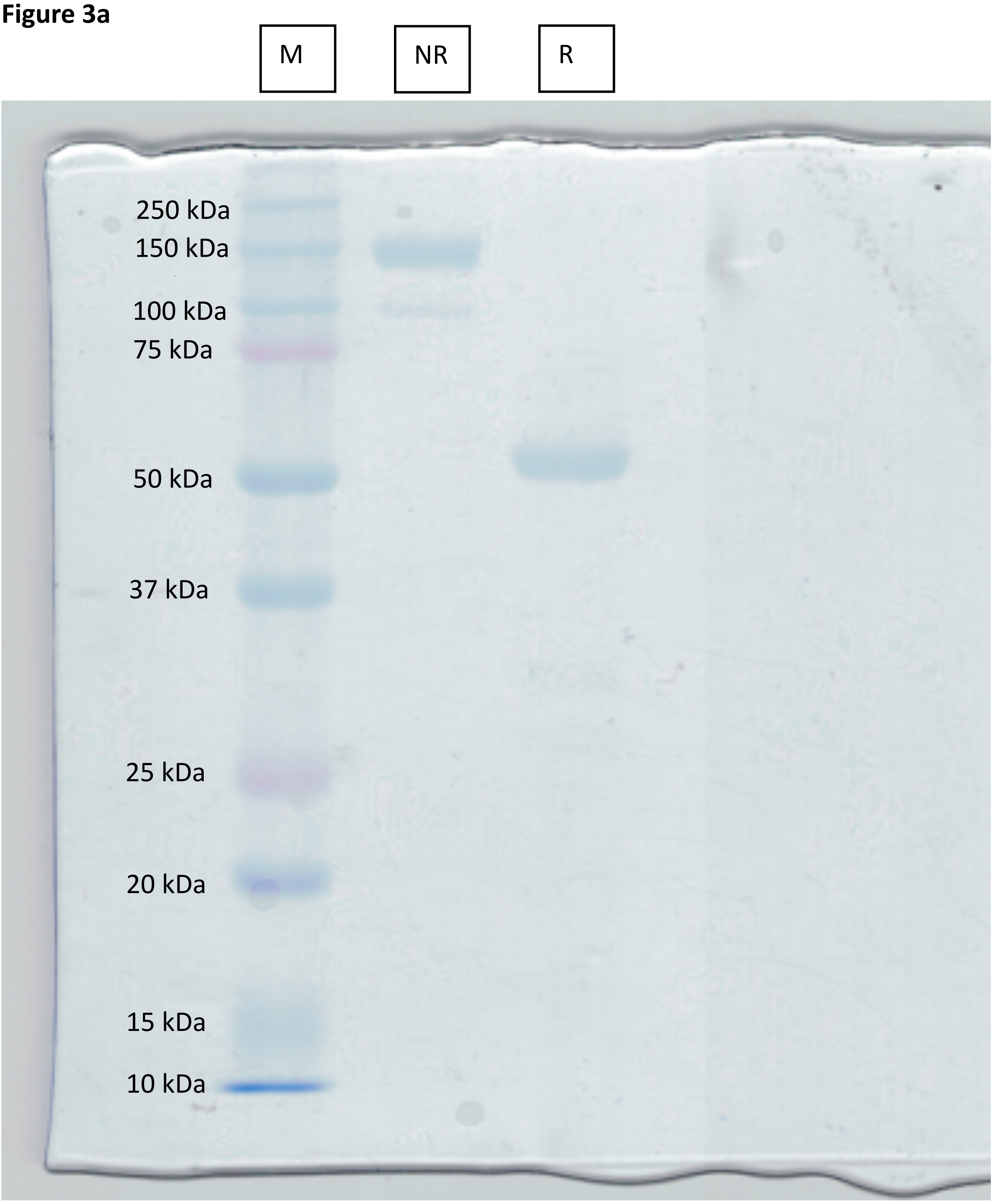


M: Marker
NR: Non-reducing condition (without DTT)
R: Reducing condition (with DTT)

**Figure 3b**

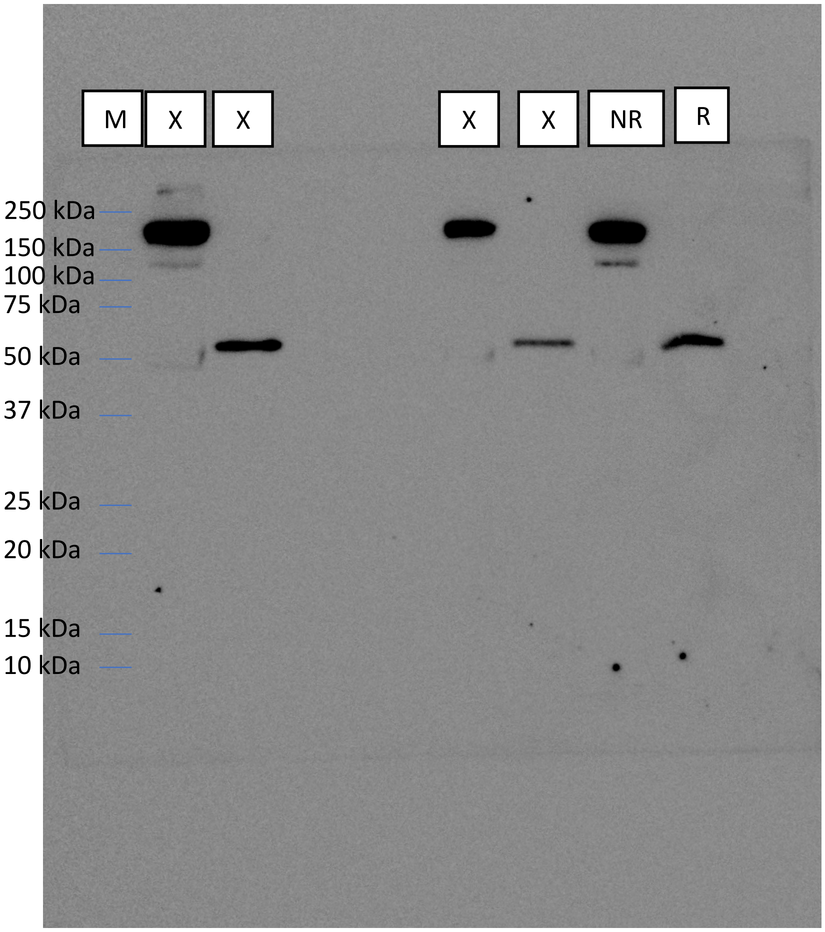


M: Marker
NR: Non-reducing condition (without DTT)
R: Reducing condition (with DTT)

**Figure 3c**

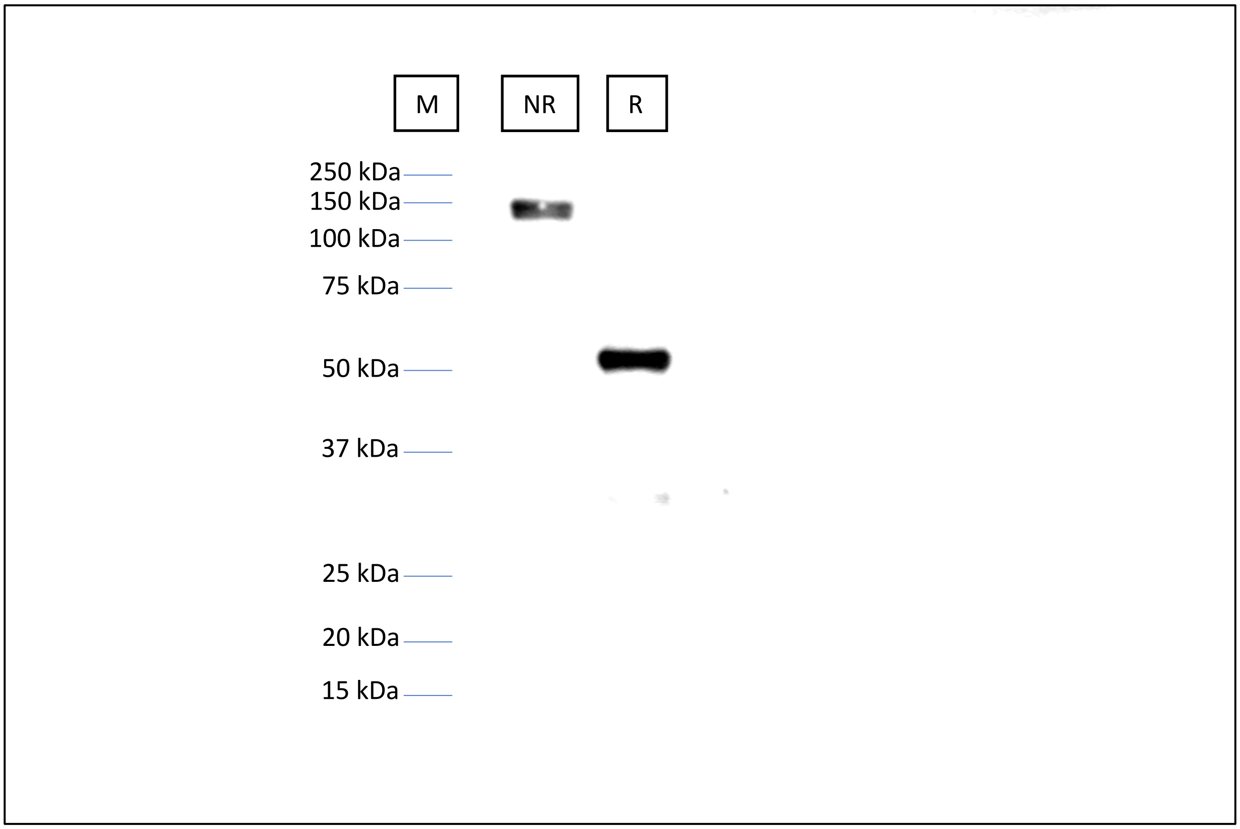


M: Marker
NR: Non-reducing condition (without DTT)
R: Reducing condition (with DTT)

**Figure 4a**

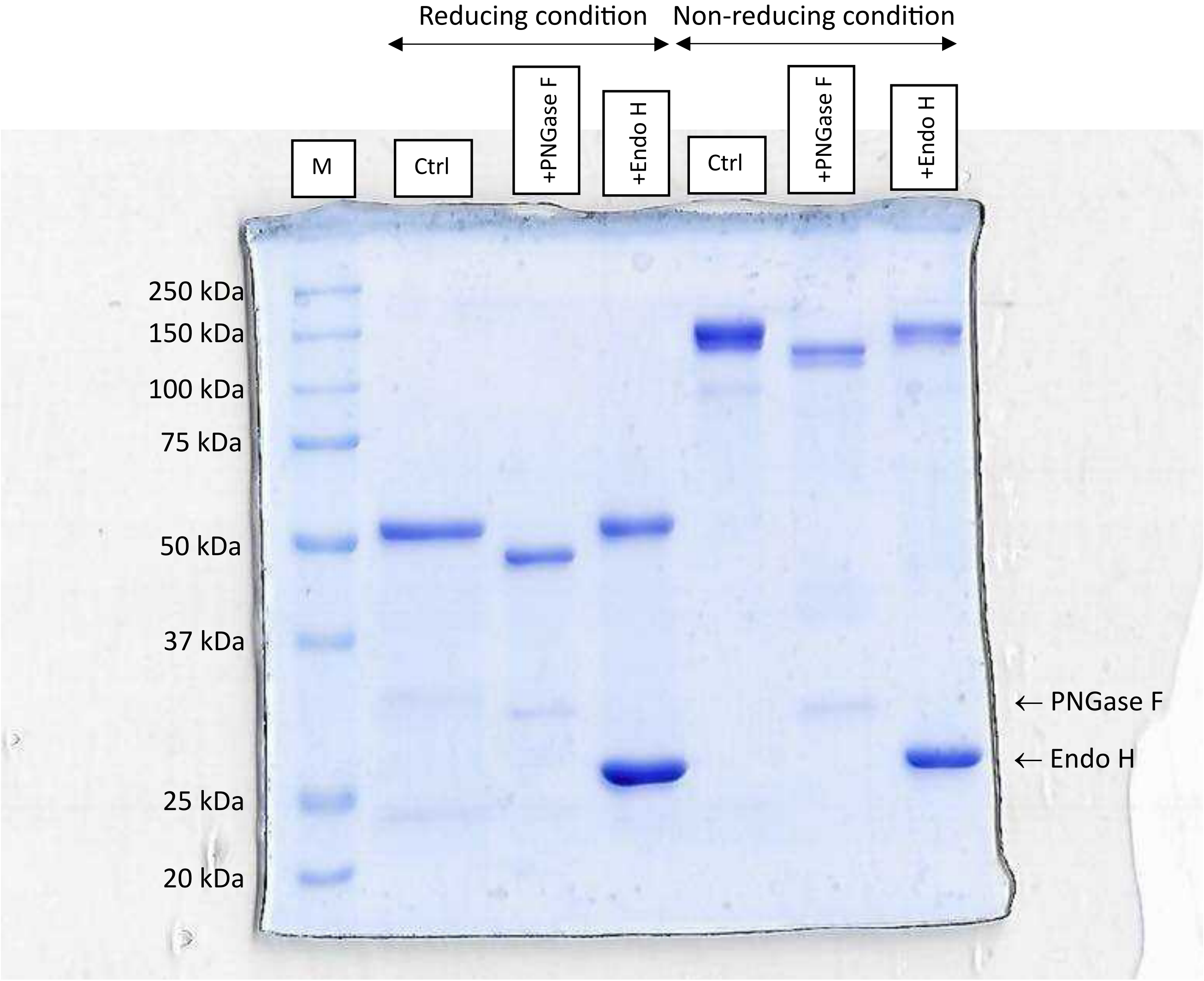


M: Marker
Ctrl: Control sample (without any enzyme digestion)
+PNGase F: Sample that treated with PNGase F
+Endo H: Sample that treated with Endo H
