# Supplementary material for: CHO-produced RBD-Fc subunit vaccines with alternative adjuvants generate immune responses against SARS-CoV-2
Source: PLoS One. 2023 Jul 14;18(7):e0288486. doi: 10.1371/journal.pone.0288486 (PMC10348575; doi:10.1371/journal.pone.0288486)
Supplement: S1 File — (DOCX) [file pone.0288486.s002.docx]

**Minimal data set: PONE-D-23-02268**

Fig. 4b

| **Sample concentration (ng/ml)** | **OD_450_** | | | | |
| --- | --- | --- | --- | --- | --- |
|  | **Replicate 1** | **Replicate 2** | **Replicate 3** | **Average** | **SD** |
| *Standard RBD-Fc (Invivogen)* | | | | | |
| 4000 | 2.916 | 2.715 | 2.995 | 2.887 | 0.1245 |
| 2000 | 2.797 | 2.640 | 2.535 | 2.657 | 0.1319 |
| 1000 | 1.873 | 1.779 | 1.703 | 1.785 | 0.0852 |
| 500 | 1.041 | 1.263 | 1.103 | 1.136 | 0.1145 |
| 250 | 0.613 | 0.586 | 0.650 | 0.616 | 0.0321 |
| 125 | 0.428 | 0.541 | 0.393 | 0.454 | 0.0773 |
| 62.50 | 0.376 | 0.387 | 0.376 | 0.380 | 0.0064 |
| 31.25 | 0.362 | 0.505^#^ | 0.382 | 0.416 | 0.0774 |
| 15.63 | 0.384 | 0.536^#^ | 1.177^#^ | 0.699 | 0.4209 |
| 7.81 | 0.344 | 0.444 | 0.378 | 0.389 | 0.0508 |
| *Our RBD-Fc* | | | | | |
| 4000 | 2.883 | 2.916 | 3.015 | 2.938 | 0.0687 |
| 2000 | 2.911 | 2.845 | 2.873 | 2.876 | 0.0331 |
| 1000 | 2.342 | 2.319 | 1.862 | 2.174 | 0.2707 |
| 500 | 1.404 | 1.407 | 1.172 | 1.328 | 0.1348 |
| 250 | 0.630 | 0.837 | 0.656 | 0.708 | 0.1128 |
| 125 | 0.591 | 0.593 | 0.451 | 0.545 | 0.0814 |
| 62.50 | 0.361 | 0.451 | 0.455 | 0.422 | 0.0532 |
| 31.25 | 0.558 | 0.333 | 0.380 | 0.424 | 0.1187 |
| 15.63 | 0.543 | 0.359 | 0.331 | 0.411 | 0.1152 |
| 7.81 | 0.441 | 0.406 | 0.334 | 0.394 | 0.0546 |
| *Negative control* | 0.322 | 0.332 | 0.309 | 0.321 | 0.0115 |

# Outliners were excluded from final calculation.

Fig. 5b

| **Sample** | **Mean of endpoint titer** | **Geometric mean of endpoint titer** | **Statistical method used** | **P value** | **No. of sample** |
| --- | --- | --- | --- | --- | --- |
|  |  |  |  |  |  |
| *Week 0* | | | | | |
| RBD-Fc + PBS | 66.67 | N/A | Kruskal-Wallis rank test in R packages. Post-hoc analyses were then performed using Dunn Kruskal-Wallis multiple comparison with Benjamini-Hochberg method p-values adjustment. | * = p<0.05  ** = p<0.01 *** = p<0.001 | 3 |
| Montanide ISA51 + PBS | 66.67 | N/A |  |  | 3 |
| RBD-Fc + Montanide ISA51 | 66.67 | N/A |  |  | 3 |
| Poly(I:C) + PBS | 33.33 | N/A |  |  | 3 |
| RBD-Fc + Poly(I:C) | 66.67 | N/A |  |  | 3 |
| MPLA/Quil-A + PBS | 66.67 | N/A |  |  | 3 |
| RBD-Fc + MPLA/Quil-A | 66.67 | N/A |  |  | 3 |
| *Week 2* | | | | | |
| RBD-Fc + PBS | 216 | 191.27 | Kruskal-Wallis rank test in R packages. Post-hoc analyses were then performed using Dunn Kruskal-Wallis multiple comparison with Benjamini-Hochberg method p-values adjustment. | * = p<0.05  ** = p<0.01 *** = p<0.001 | 5 |
| Montanide ISA51 + PBS | 140 | 131.95 |  |  | 5 |
| RBD-Fc + Montanide ISA51 | 2160 | 1912.70 |  |  | 5 |
| Poly(I:C) + PBS | 100 | N/A |  |  | 5 |
| RBD-Fc + Poly(I:C) | 1640 | 1098.56 |  |  | 5 |
| MPLA/Quil-A + PBS | 60 | N/A |  |  | 5 |
| RBD-Fc + MPLA/Quil-A | 1440 | 1098.56 |  |  | 5 |
| *Week 5* | | | | | |
| RBD-Fc + PBS | 19600 | 7282.26 | Kruskal-Wallis rank test in R packages. Post-hoc analyses were then performed using Dunn Kruskal-Wallis multiple comparison with Benjamini-Hochberg method p-values adjustment. | * = p<0.05  ** = p<0.01 *** = p<0.001 | 5 |
| Montanide ISA51 + PBS | 400 | 263.90 |  |  | 5 |
| RBD-Fc + Montanide ISA51 | 640000 | 606286.63 |  |  | 5 |
| Poly(I:C) + PBS | 220 | N/A |  |  | 5 |
| RBD-Fc + Poly(I:C) | 416000 | 333021.28 |  |  | 5 |
| MPLA/Quil-A + PBS | 240 | N/A |  |  | 5 |
| RBD-Fc + MPLA/Quil-A | 200000 | 132578.16 |  |  | 5 |
| *Week 8* | | | | | |
| RBD-Fc + PBS | 15200 | 13862.90 | Kruskal-Wallis rank test in R packages. Post-hoc analyses were then performed using Dunn Kruskal-Wallis multiple comparison with Benjamini-Hochberg method p-values adjustment. | * = p<0.05  ** = p<0.01 *** = p<0.001 | 5 |
| Montanide ISA51 + PBS | 240 | 231.42 |  |  | 5 |
| RBD-Fc + Montanide ISA51 | 520000 | 459479.34 |  |  | 5 |
| Poly(I:C) + PBS | 240 | 231.42 |  |  | 5 |
| RBD-Fc + Poly(I:C) | 360000 | 348220.23 |  |  | 5 |
| MPLA/Quil-A + PBS | 180 | 175.08 |  |  | 5 |
| RBD-Fc + MPLA/Quil-A | 280000 | 263901.58 |  |  | 5 |

Fig. 5c

| **Sample** | **Mean of endpoint titer** | **Geometric mean of endpoint titer** | **Statistical method used** | **P value** | **No. of sample** |  |  |
| --- | --- | --- | --- | --- | --- | --- | --- |
|  |  |  |  |  |  |  |  |
| *Week 0* | | | | | |  |  |
| RBD-Fc + PBS | 0 | N/A | Kruskal-Wallis rank test in R packages. Post-hoc analyses were then performed using Dunn Kruskal-Wallis multiple comparison with Benjamini-Hochberg method p-values adjustment. | * = p<0.05  ** = p<0.01 *** = p<0.001 | 3 |  |  |
| Montanide ISA51 + PBS | 0 | N/A |  |  | 3 |  |  |
| RBD-Fc + Montanide ISA51 | 0 | N/A |  |  | 3 |  |  |
| Poly(I:C) + PBS | 0 | N/A |  |  | 3 |  |  |
| RBD-Fc + Poly(I:C) | 0 | N/A |  |  | 3 |  |  |
| MPLA/Quil-A + PBS | 0 | N/A |  |  | 3 |  |  |
| RBD-Fc + MPLA/Quil-A | 0 | N/A |  |  | 3 |  |  |
| *Week 2* | | | | | |  |  |
| RBD-Fc + PBS | 208 | 166.51 | Kruskal-Wallis rank test in R packages. Post-hoc analyses were then performed using Dunn Kruskal-Wallis multiple comparison with Benjamini-Hochberg method p-values adjustment. | * = p<0.05  ** = p<0.01 *** = p<0.001 | 5 |  |  |
| Montanide ISA51 + PBS | 0 | N/A |  |  | 5 |  |  |
| RBD-Fc + Montanide ISA51 | 5200 | 4594.79 |  |  | 5 |  |  |
| Poly(I:C) + PBS | 0 | N/A |  |  | 5 |  |  |
| RBD-Fc + Poly(I:C) | 2960 | 2197.12 |  |  | 5 |  |  |
| MPLA/Quil-A + PBS | 0 | N/A |  |  | 5 |  |  |
| RBD-Fc + MPLA/Quil-A | 2560 | 2197.12 |  |  | 5 |  |  |
| *Week 5* | | | | | |  |  |
| RBD-Fc + PBS | 22600 | 8746.90 | Kruskal-Wallis rank test in R packages. Post-hoc analyses were then performed using Dunn Kruskal-Wallis multiple comparison with Benjamini-Hochberg method p-values adjustment. | * = p<0.05  ** = p<0.01 *** = p<0.001 | 5 |  |  |
| Montanide ISA51 + PBS | 0 | N/A |  |  | 5 |  |  |
| RBD-Fc + Montanide ISA51 | 640000 | 551891.86 |  |  | 5 |  |  |
| Poly(I:C) + PBS | 0 | N/A |  |  | 5 |  |  |
| RBD-Fc + Poly(I:C) | 200000 | 200000 |  |  | 5 |  |  |
| MPLA/Quil-A + PBS | 0 | N/A |  |  | 5 |  |  |
| RBD-Fc + MPLA/Quil-A | 300000 | 263901.58 |  |  | 5 |  |  |
| *Week 8* | | | | | |  |  |
| RBD-Fc + PBS | 252000 | 171902.98 | Kruskal-Wallis rank test in R packages. Post-hoc analyses were then performed using Dunn Kruskal-Wallis multiple comparison with Benjamini-Hochberg method p-values adjustment. | * = p<0.05  ** = p<0.01 *** = p<0.001 | 5 |  |  |
| Montanide ISA51 + PBS | 0 | N/A |  |  | 5 |  |  |
| RBD-Fc + Montanide ISA51 | 1240000 | 1080036.94 |  |  | 5 |  |  |
| Poly(I:C) + PBS | 0 | N/A |  |  | 5 |  |  |
| RBD-Fc + Poly(I:C) | 400000 | 400000 |  |  | 5 |  |  |
| MPLA/Quil-A + PBS | 0 | N/A |  |  | 5 |  |  |
| RBD-Fc + MPLA/Quil-A | 440000 | 406404.75 |  |  | 5 |  |  |

Fig. 5d

| **Sample** | **Mean of endpoint titer** | **Geometric mean of endpoint titer** | **Statistical method used** | **P value** | **No. of sample** |  |  |
| --- | --- | --- | --- | --- | --- | --- | --- |
|  |  |  |  |  |  |  |  |
| *Week 0* | | | | | |  |  |
| RBD-Fc + PBS | 0 | N/A | Kruskal-Wallis rank test in R packages. Post-hoc analyses were then performed using Dunn Kruskal-Wallis multiple comparison with Benjamini-Hochberg method p-values adjustment. | * = p<0.05  ** = p<0.01 *** = p<0.001 | 3 |  |  |
| Montanide ISA51 + PBS | 0 | N/A |  |  | 3 |  |  |
| RBD-Fc + Montanide ISA51 | 0 | N/A |  |  | 3 |  |  |
| Poly(I:C) + PBS | 0 | N/A |  |  | 3 |  |  |
| RBD-Fc + Poly(I:C) | 0 | N/A |  |  | 3 |  |  |
| MPLA/Quil-A + PBS | 0 | N/A |  |  | 3 |  |  |
| RBD-Fc + MPLA/Quil-A | 0 | N/A |  |  | 3 |  |  |
| *Week 2* | | | | | |  |  |
| RBD-Fc + PBS | 0 | N/A | Kruskal-Wallis rank test in R packages. Post-hoc analyses were then performed using Dunn Kruskal-Wallis multiple comparison with Benjamini-Hochberg method p-values adjustment. | * = p<0.05  ** = p<0.01 *** = p<0.001 | 5 |  |  |
| Montanide ISA51 + PBS | 0 | N/A |  |  | 5 |  |  |
| RBD-Fc + Montanide ISA51 | 20 | N/A |  |  | 5 |  |  |
| Poly(I:C) + PBS | 0 | N/A |  |  | 5 |  |  |
| RBD-Fc + Poly(I:C) | 200 | N/A |  |  | 5 |  |  |
| MPLA/Quil-A + PBS | 0 | N/A |  |  | 5 |  |  |
| RBD-Fc + MPLA/Quil-A | 520 | N/A |  |  | 5 |  |  |
| *Week 5* | | | | | |  |  |
| RBD-Fc + PBS | 240 | N/A | Kruskal-Wallis rank test in R packages. Post-hoc analyses were then performed using Dunn Kruskal-Wallis multiple comparison with Benjamini-Hochberg method p-values adjustment. | * = p<0.05  ** = p<0.01 *** = p<0.001 | 5 |  |  |
| Montanide ISA51 + PBS | 0 | N/A |  |  | 5 |  |  |
| RBD-Fc + Montanide ISA51 | 8000 | 7614.62 |  |  | 5 |  |  |
| Poly(I:C) + PBS | 0 | N/A |  |  | 5 |  |  |
| RBD-Fc + Poly(I:C) | 28000 | 20000 |  |  | 5 |  |  |
| MPLA/Quil-A + PBS | 0 | N/A |  |  | 5 |  |  |
| RBD-Fc + MPLA/Quil-A | 46000 | 34822.02 |  |  | 5 |  |  |
| *Week 8* | | | | | |  |  |
| RBD-Fc + PBS | 80 | N/A | Kruskal-Wallis rank test in R packages. Post-hoc analyses were then performed using Dunn Kruskal-Wallis multiple comparison with Benjamini-Hochberg method p-values adjustment. | * = p<0.05  ** = p<0.01 *** = p<0.001 | 5 |  |  |
| Montanide ISA51 + PBS | 0 | N/A |  |  | 5 |  |  |
| RBD-Fc + Montanide ISA51 | 18000 | 17411.01 |  |  | 5 |  |  |
| Poly(I:C) + PBS | 0 | N/A |  |  | 5 |  |  |
| RBD-Fc + Poly(I:C) | 60000 | 50237.73 |  |  | 5 |  |  |
| MPLA/Quil-A + PBS | 0 | N/A |  |  | 5 |  |  |
| RBD-Fc + MPLA/Quil-A | 56000 | 52780.32 |  |  | 5 |  |  |

Fig. 7

| **Sample** | **Mean of PRNT_50_** | **Geometric mean of PRNT_50_** | **Mean of PRNT_90_** | **Geometric mean of PRNT_90_** | **Statistical method used** | **P value** | **No. of sample** |
| --- | --- | --- | --- | --- | --- | --- | --- |
| *Week 5* | | | | | | | |
| RBD-Fc + PBS | N/A | N/A | N/A | N/A | Kruskal-Wallis rank test in R packages. Post-hoc analyses were then performed using Dunn Kruskal-Wallis multiple comparison with Benjamini-Hochberg method p-values adjustment. | * = p<0.05  ** = p<0.01 | 5 |
| RBD-Fc + Montanide ISA51 | 3574 | 2983.40 | 2052 | 1715.05 |  |  | 5 |
| RBD-Fc + Poly(I:C) | 2040 | 1721.99 | 1172 | 989.72 |  |  | 5 |
| RBD-Fc + MPLA/Quil-A | 1344 | 1190.72 | 772 | 683.94 |  |  | 5 |
| *Week 8* | | | | | | | |
| RBD-Fc + PBS | N/A | N/A | N/A | N/A | Kruskal-Wallis rank test in R packages. Post-hoc analyses were then performed using Dunn Kruskal-Wallis multiple comparison with Benjamini-Hochberg method p-values adjustment. | * = p<0.05  ** = p<0.01 | 5 |
| RBD-Fc + Montanide ISA51 | N/A | N/A | 4614 | 4110.68 |  |  | 5 |
| RBD-Fc + Poly(I:C) | N/A | N/A | 3946 | 3794.22 |  |  | 5 |
| RBD-Fc + MPLA/Quil-A | N/A | N/A | 2738 | 2556.09 |  |  | 5 |

Fig. 8

| **Sample** | **Mean of sVNT_50_** | **Geometric mean of sVNT_50_** | **Statistical method used** | **P value** | **No. of sample** |
| --- | --- | --- | --- | --- | --- |
| *Week 8* | | | | | |
| RBD-Fc + PBS | 127.11 | 48.19 | Kruskal-Wallis rank test in R packages. Post-hoc analyses were then performed using Dunn Kruskal-Wallis multiple comparison with Benjamini-Hochberg method p-values adjustment. | * = p<0.05  ** = p<0.01 | 5 |
| RBD-Fc + Montanide ISA51 | 43623.24 | 24416.05 |  |  | 5 |
| RBD-Fc + Poly(I:C) | 6973.17 | 6285.02 |  |  | 5 |
| RBD-Fc + MPLA/Quil-A | 6268.41 | 4980.81 |  |  | 5 |

*#N/A* means the value cannot be calculated because some of individual data is equal to zero or raw data is not exact number (exceeding detection range).
